# Combining *in vivo* proton exchange rate (*k*_ex_) MRI with quantitative susceptibility mapping to further stratify the gadolinium-negative multiple sclerosis lesions

**DOI:** 10.3389/fnins.2022.1105376

**Published:** 2023-01-11

**Authors:** Huiting Liao, Zimeng Cai, Haiqi Ye, QianLan Chen, Yan Zhang, Mehran Shaghaghi, Sarah E. Lutz, Weiwei Chen, Kejia Cai

**Affiliations:** ^1^Department of Radiology, Tongji Hospital, Tongji Medical College, Huazhong University of Science and Technology, Wuhan, China; ^2^Department of Radiology, Guangdong Provincial People’s Hospital, Guangdong Academy of Medical Sciences, Guangzhou, China; ^3^Guangdong Provincial Key Laboratory of Artificial Intelligence in Medical Image Analysis and Application, Guangdong Provincial People’s Hospital, Guangdong Academy of Medical Sciences, Guangzhou, China; ^4^Department of Radiology, Hangzhou First People’s Hospital, Zhejiang University School of Medicine, Hangzhou, China; ^5^Experimental and Clinical Research Center, Max Delbrück Center for Molecular Medicine and Charité – Universitätsmedizin Berlin, Corporate Member of Freie Universität Berlin, Berlin Institute of Health, Humboldt-Universität zu Berlin, Berlin, Germany; ^6^Department of Radiology, University of Illinois Hospital and Health Sciences System, Chicago, IL, United States; ^7^Department of Anatomy and Cell Biology, University of Illinois at Chicago College of Medicine, Chicago, IL, United States

**Keywords:** multiple sclerosis, magnetic resonance imaging, oxidative stress, iron, inflammation

## Abstract

**Background:**

Conventional gadolinium (Gd)-enhanced MRI is currently used for stratifying the lesion activity of multiple sclerosis (MS) despite limited correlation with disability and disease activity. The stratification of MS lesion activity needs further improvement to better support clinics.

**Purpose:**

To investigate if the novel proton exchange rate (*k*_*ex*_) MRI combined with quantitative susceptibility mapping (QSM) may help to further stratify non-enhanced (Gd-negative) MS lesions.

**Materials and methods:**

From December 2017 to December 2020, clinically diagnosed relapsing-remitting MS patients who underwent MRI were consecutively enrolled in this IRB-approved retrospective study. The customized MRI protocol covered conventional T_2_-weighted, T_2_-fluid-attenuated-inversion-recovery, pre- and post-contrast T_1_-weighted imaging, and quantitative sequences, including *k*_*ex*_ MRI based on direct-saturation removed omega plots and QSM. Each MS lesion was evaluated based on its Gd-enhancement as well as its susceptibility and *k*_*ex*_ elevation compared to the normal appearing white matter. The difference and correlation concerning lesion characteristics and imaging contrasts were analyzed using the Mann–Whitney U test or Kruskal–Wallis test, and Spearman rank analysis with *p* < 0.05 considered significant.

**Results:**

A total of 322 MS lesions from 30 patients were identified with 153 Gd-enhanced and 169 non-enhanced lesions. We found that the *k*_*ex*_ elevation of all lesions significantly correlated with their susceptibility elevation (*r* = 0.30, *p* < 0.001). Within the 153 MS lesions with Gd-enhancement, ring-enhanced lesions showed higher *k*_*ex*_ elevation than the nodular-enhanced ones’ (*p* < 0.001). Similarly, lesions with ring-hyperintensity in QSM also had higher *k*_*ex*_ elevation than the lesions with nodular-QSM-hyperintensity (*p* < 0.001). Of the 169 Gd-negative lesions, three radiological patterns were recognized according to lesion manifestations on the *k*_*ex*_ map and QSM images: Pattern I (*k*_*ex*_^+^ and QSM^+^, *n* = 114, 67.5%), Pattern II (only *k*_*ex*_^+^ or QSM^+^, *n* = 47, 27.8%) and Pattern III (*k*_*ex*_^–^ and QSM^–^, *n* = 8, 4.7%). Compared to Pattern II and III, Pattern I had higher *k*_*ex*_ (*p* < 0.001) and susceptibility (*p* < 0.05) elevation. The percentage of Pattern I of each subject was negatively correlated with the disease duration (*r* = –0.45, *p* = 0.015).

**Conclusion:**

As a potential imaging biomarker for inflammation due to oxidative stress, *in vivo k*_*ex*_ MRI combined with QSM is promising in extending the clinical classification of MS lesions beyond conventional Gd-enhanced MRI.

## Introduction

Multiple sclerosis (MS) is a chronic inflammatory demyelinating and neurodegenerative disorder of the central nervous system. Ongoing inflammation causes neuronal damage in MS patients (Kotelnikova et al., 2017). Clinical, radiological, and histological classification systems have been proposed (Pitt et al., 2022). Histological evidence of inflammation is incorporated to categorize lesions into early active, late active, smoldering, inactive, and shadow plaques (Frischer et al., 2015). Refinement of these classification systems to incorporate recently developed radiologic techniques for non-invasive evidence of inflammation may further guide clinical management and elucidation of disease pathogenesis.

To date, a commonly recognized classification in the clinic is to differentiate active (acute) from inactive lesions. Introducing gadolinium (Gd)-based contrast agents, Gd contrast-enhanced MRI can identify acute MS lesions (Tourdias et al., 2012), because the inflammatory infiltration led by lymphocytes and microglia can affect blood-brain-barrier (BBB) functions, allowing extravasation of gadolinium-based contrast agents (Lassmann et al., 2007). However, the gadolinium enhancement of acute lesions usually only lasts for weeks (Mahad et al., 2015). There is evidence suggesting that the gadolinium-enhanced lesions would turn into non-gadolinium-enhanced lesions even with inflammation continuously proceeding (Maggi et al., 2014). Moreover, the potential risk of Gd accumulation requires clinicians to make judicious use of this imaging method (Wattjes et al., 2021). Clinical practitioners may be able to make more personalized treatments and monitor the therapeutic efficacy of treatment by reconsidering the interpretation of lesion activity, especially for Gd non-enhanced (Gd-negative) lesions.

There has been a significant amount of research dedicated to the recognition of smoldering lesions or detecting inflammatory changes in MS lesions. Quantitative susceptibility mapping (QSM) could reflect iron deposition when the relative lesion susceptibility value is above zero (Wisnieff et al., 2015). The susceptibility of MS lesions increases significantly when gadolinium-enhanced lesions evolve into non-gadolinium-enhanced lesions (Zhang et al., 2016b). In addition to iron, reactive oxygen species (ROS) are also reported to be key elements of inflammation (Dunham et al., 2017). The proton exchange rate (*k*_*ex*_) MRI approach (Shaghaghi et al., 2019), derived from chemical exchange saturation transfer (CEST) imaging, has recently been proposed as a non-invasive approach to assess tissue oxidative stress since endogenous ROS have been shown to promote *in vivo k*_*ex*_ in tissue (Tain et al., 2019; Shaghaghi and Cai, 2022). Our team has previously conducted a preliminary study on *k*_*ex*_ MRI showing its potential to further characterize Gd-negative MS lesions (Ye et al., 2020). However, the contrast mechanism of *k*_*ex*_-enhanced MRI as a surrogate biomarker for ROS has not been fully validated. Given that iron deposition catalyzes Fenton reaction that produces hydroxyl ROS (Tain et al., 2019), the correlation between QSM and *k*_*ex*_ MRI may help to validate *k*_*ex*_ MRI as an emerging inflammatory imaging contrast for MS stratification.

In the present study, we will compare *k*_*ex*_ MRI with the relatively well-established QSM in all MS lesions and investigate if the combination of *k*_*ex*_ MRI and QSM helps to stratify Gd-negative MS lesions based on lesion inflammatory activity.

## Materials and methods

### Participants

From December 2017 to December 2020, patients with relapsing-remitting MS in our institution who underwent MRI were consecutively included in the following institutional review board-approved retrospective study. All patients were clinically diagnosed according to the 2017 revision of the McDonald criteria (Thompson et al., 2018a). Cases with large motion artifacts were excluded. Relevant clinical information including major symptoms and Kurtzke Expanded Disability Status Scale (EDSS) scores was recorded. The onset date was also recorded according to the patients’ questionnaire. The disease duration of each patient was estimated from the onset date to the day of receiving the MRI.

### Image acquisition

All MRI exams were performed on a 3T GE MR750 unit (GE Healthcare, Milwaukee, WI) with a 32-channel head coil using a customized MRI protocol, including T_2_-weighted imaging, T_2_ fluid-attenuated inversion recovery (T_2_-FLAIR) imaging, T_2_*-weighted angiography for the QSM (Chen et al., 2014), CEST sequences for the *k*_*ex*_ MRI (Shaghaghi et al., 2019), and pre- and post-contrast T_1_-weighted imaging. All sequences within the protocols were performed with matched slice positioning to allow lesion comparison between different MRI sequences. Post-contrast T_1_-weighted imaging scans started 5 min after contrast agent administration (gadopentetate dimeglumine, Magnevist, Bayer, Berlin, Germany, 0.1 mmol/kg, IV).

The *k*_*ex*_ MRI protocol comprised three CEST Z-spectral data acquired with different saturation powers (B_1_) at 2, 3, and 4 μT and a saturation duration of 1.5 s, followed by a single-shot fast low angle shot readout. The total acquisition time of the three Z-spectra used for constructing omega plots was 9.9 min. At each saturation B_1_, a total of 33 frequency offsets were obtained, including +39.1 ppm, +15.6 ppm, ±6 ppm, ±5 ppm, ±4.5 ppm, and frequencies ranging from –4 to +4 ppm with an increment of 0.25 ppm. CEST Z-spectral data were all acquired from a single slice covering most MS lesions as delineated by the T_2_-FLAIR images. The target slice of each patient was decided by the same neuroradiologist (Q.C., 4 years of experience in neuroradiology). The detailed parameters of each sequence are summarized in Table 1.

**TABLE 1:** Detailed parameters of each sequence.

Parameter	T1*	T2^†^	T2 FLAIR^‡^	*k*_ex_ MRI^§^	ESWAN^θ^
Repetition time (msec)	500	5300	8400	3000	57
Effective echo time (msec)	8	92	145	22.6	4.3/4.8**
Slice thickness (mm)	5	5	5	5	2
Field of view (cm)	24	24	24	24	24
Matrix	320 × 320	512 × 512	512 × 512	128 × 128	512 × 512

*Pre- and post-contrast axial T_1_-weighted fast spin-echo sequences (contrast agent: gadopentetate dimeglumine, Magnevist, Bayer, Berlin, Germany, 0.1 mmol/kg, IV).

^†^Axial T_2_-weighted fast spin-echo sequences.

^‡^Axial T_2_ fluid-attenuated inversion recovery sequences.

^§^ Proton exchange rate MRI based on chemical exchange saturation transfer sequence.

^θ^Three-dimensional eight-echo T2*-weighted angiography.

**First echo time/echo time spacing.

### Image reconstruction

Quantitative susceptibility mapping was reconstructed based on the morphology enabled dipole inversion (MEDI) algorithm using in-house software implemented in C++ (Liu et al., 2012). The reconstruction was automatic without any user intervention. All acquired Z-spectral data were analyzed with the direct-saturation removed omega plot method (Shaghaghi et al., 2019) using MATLAB (MathWorks, Natick, MA, USA) with custom-written scripts. Equations were generated to reflect multiple exchange mechanisms, including NOE, MT, and CEST, that contribute to the saturation transfer signal (Shaghaghi et al., 2019). The reference image (39.1 ppm) from each CEST sequence was registered to that of B_1_ = 3 μT sequence and each image within each CEST sequence was registered to its previous adjacent image. Z-spectra were normalized, flipped and fitted to two Lorentzian functions corresponding to the bulk water (centered around 0 ppm) and to a sum of the remaining effects (centered around 1.5 ppm). The frequency offset relative to water resonance, the center frequency offset, the amplitude, and the line-width of each peak were considered in the fitting function. After the fitting, the bulk water peak-the dominant component-was subtracted from the raw Z-spectra and the B_0_-corrected residual signals at +3.5 ppm were used for further omega plot analysis. Tissue *k*_*ex*_ is a weighted average of multiple exchange mechanisms. With the *k*_*ex*_ quantified for every voxel, *k*_*ex*_ maps were reconstructed.

### Classification of MS lesions

Lesions with T2 hyperintensities (at least 3 mm on the long axis) were considered MS lesions in this study (Filippi et al., 2016). White matter regions without an abnormal signal were regarded as normal-appearing white matter (NAWM). Three neuroradiologists (H.L., Q.C., W.C., with 2, 4, and 20 years of experience in neuroradiology, respectively) independently reviewed all MRI images. The three observers assessed all MS lesions according to the presence of Gd enhancement, QSM hyperintensity, and *k*_*ex*_ elevation, as well as the lesion shape (nodular or ring) of Gd enhancement and QSM hyperintensity.

On post-contrast images, MS lesions were identified as enhanced (Gd-positive, Gd^+^) or non-enhanced (Gd-negative, Gd^–^). The Gd^+^ lesions were further classified as nodular-enhanced or ring-enhanced based on their shape. Lesions without QSM hyperintensity were considered QSM negative (QSM^–^), while lesions with QSM hyperintensity (QSM^+^) were also further categorized as nodular-QSM-hyperintense or ring-QSM-hyperintense (hyperintensity at the edge of the lesion). On the *k*_*ex*_ maps, we classified lesions into two groups: one without *k*_*ex*_ elevation (*k*_*ex*_^–^) and another with *k*_*ex*_ elevation (*k*_*ex*_^+^) relative to its adjacent or contralateral NAWM area. No further classification was performed based on the shape of the *k*_*ex*_^+^ lesion, as the *k*_*ex*_ elevation within each lesion was relatively uniform. Interobserver agreement for lesion classification was assessed and any difference was discussed, and a consensus was reached for further quantitative analyses.

### Measurement of lesion *k*_*ex*_ elevation and susceptibility elevation

The *k*_*ex*_ elevation (Δ*k*_*ex*_) or susceptibility elevation (ΔSusceptibility) of a lesion was defined as the difference of *k*_*ex*_ or susceptibility value between the lesion and its contralateral or adjacent NAWM area. The ROIs of *k*_*ex*_ and susceptibility were drawn using an in-house ROI tool of ITK-SNAP software (v4.0.0, Yushkevich et al., 2006) by two neuroradiologists (H.L. and Q.C.) independently. Taking T_2_-FLAIR and T_2_-weighted images as a reference, the lesion ROIs were drawn on the *k*_*ex*_ map and QSM images by hand, outlining the boundaries of each identified MS lesion. For inconspicuous lesions on the *k*_*ex*_ map or QSM images, the ROIs were first manually drawn on T_2_-FLAIR images (or T_2_-weighted images when they showed clear lesion boundaries) and then overlaid onto the *k*_*ex*_ map and QSM images. All veins or artifacts within lesions on QSM were also removed by hand. The ROIs of NAWM (rectangle, 4 mm^2^) were also placed on the mirror-symmetric area (or the adjacent NAWM of MS lesions if the mirror-symmetric area happened to be another lesion) as an internal reference. Interobserver agreement analysis was performed and the mean results of the two observers were used for further analyses.

### Statistical analysis

Statistical analyses were performed by GraphPad Prism (v9.4.1) and SPSS (v26.0, IBM). Continuous variables were presented as mean ± standard deviation, while categorical results were presented as fractions or percentages. Interobserver agreement between radiologists was measured by Fleiss’ Kappa statistic (for the presence and shape of the lesion) or the intraclass correlation coefficient (for lesion measurement). The Kolmogorov–Smirnov test was used to determine the normality of the data. The comparison of lesion *k*_*ex*_ elevation or susceptibility elevation in subgroups was analyzed *via* the two-tailed Student’s *t*-test and one-way ANOVA (Gaussian distribution) or Mann–Whitney U test and Kruskal–Wallis test (non-Gaussian distribution). The Spearman rank analysis were used to explore differences and correlations concerning lesion characteristics in different images. The statistical significance level, *p*, was set at 0.05.

## Results

### Demographic and clinical features

From December 2017 to December 2020, 34 patients with relapsing-remitting MS were identified and a total of 48 MRI exams were retrieved from the institutional database as 6 of the 34 patients had one or more follow-up scans. Seven MRI exams from four patients were excluded for the failure of image reconstruction caused by motion artifacts. Ultimately, 30 patients and a total of 41 MRI examinations were included in the analysis, including data from five patients who had one or more follow-up exams. There were 21 women and 9 men. Their age ranged from 18 to 54 (mean ± SD = 31.30 ± 9.73) years old. The mean disease duration was 3.86 ± 4.77 years (ranging from 0.05 to 20 years), with a median expanded disability status scale of 2.0 (ranging from 1.0 to 8.5). Demographic and clinical information with details are given in Supplementary Table 1.

### Lesion manifestation on QSM, *k*_*ex*_, and post-contrast MRI

**FIGURE 1:**
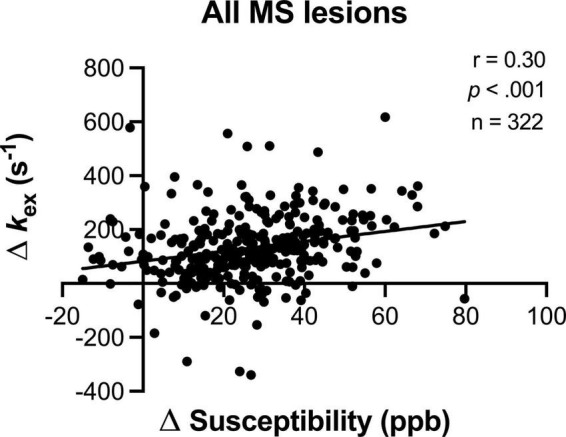
The lesion *k*_*ex*_ elevation increased with susceptibility elevation. Note that a few lesions that showed negative Δ*k*_*ex*_ were highly necrotic as confirmed with T_2_-weighted and T_2_-FLAIR imaging.

There was high agreement among observers in the assessment of the lesion presence, the lesion shape (Fleiss’ Kappa coefficient > 0.800 for both), and the quantitative measurement (intraclass correlation coefficient > 0.800) (Supplementary Table 2). A total of 322 MS lesions were identified. The number of lesions with *k*_*ex*_ elevation (*n* = 261) or with QSM hyperintensity (*n* = 282) was larger than that with Gd-enhancement (*n* = 153). The *k*_*ex*_ elevation of MS lesions were positively and significantly correlated with the lesions’ susceptibilities elevation (*r* = 0.30, *p* < 0.001, Figure 1). Note that a few lesions that showed negative Δ*k*_*ex*_ in Figure 1 were highly necrotic as confirmed with T_2_-weighted and T_2_-FLAIR imaging.

### QSM and *k*_*ex*_ features in Gd^+^ lesions

**FIGURE 2:**
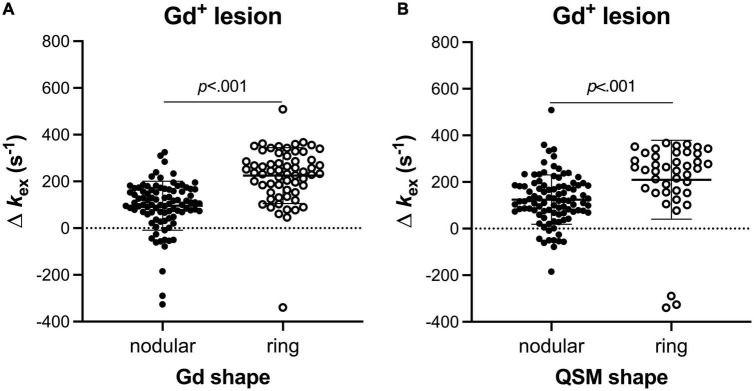
Different Gd^+^ MS lesions showed different levels of *k*_*ex*_ values (Mann–Whitney U test). Lesions with ring Gd-enhancement presented higher *k*_*ex*_ elevation than those with nodular Gd-enhancements **(A)**. For the lesions being both positive on post-contrast T1 imaging and QSM, the ones with ring QSM hyperintensity presented higher *k*_*ex*_ elevation than those with nodular QSM hyperintensity **(B)**.

In total, 153 MS lesions were Gd^+^. These Gd^+^ lesions showed two types of enhancement: ring-enhanced (61/153, 39.9%) and nodular-enhanced (92/153, 60.1%). The ring-enhanced ones presented higher *k*_*ex*_ elevation than the nodular-enhanced ones (225.0 ± 119.2 s^–1^ vs. 95.7 ± 104.8 s^–1^, *p* < 0.001, Figure 2A). On QSM, 89.5% (137/153) of the Gd^+^ lesions showed hyperintensity with two different shapes: nodular and ring-like. The lesions with ring-like QSM hyperintensity presented higher *k*_*ex*_ elevation than those with nodular QSM hyperintensity (209.7 ± 169.6 s^–1^ vs. 124.5 ± 105.6 s^–1^, *p* < 0.001, Figure 2B).

### QSM and *k*_*ex*_ features in Gd^–^ MS lesions

**FIGURE 3:**
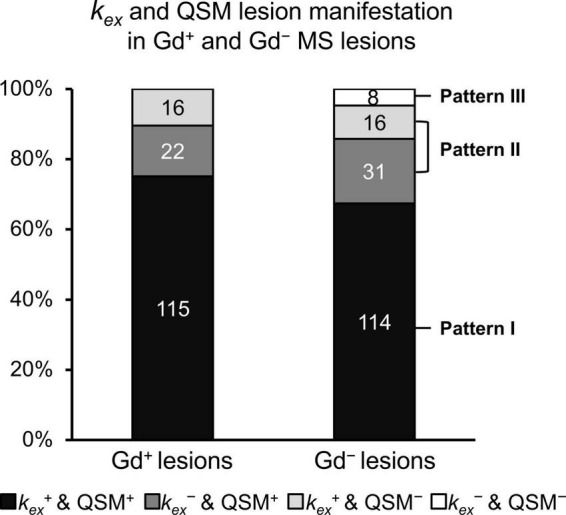
Extended classification of Gd^–^ lesions. Gd^–^ lesions were categorized into Pattern I (which are *k*_*ex*_^+^ and QSM^+^); Pattern II (which are either *k*_*ex*_^+^ and QSM^–^, or *k*_*ex*_^–^ and QSM^+^); and Pattern III (which are *k*_*ex*_^–^ and QSM^–^) according to lesion manifestation on the *k*_*ex*_ map and QSM images. All Gd^+^ lesions were considered as one category despite the inconsistent lesion manifestation on the *k*_*ex*_ map and QSM images.

**FIGURE 4:**
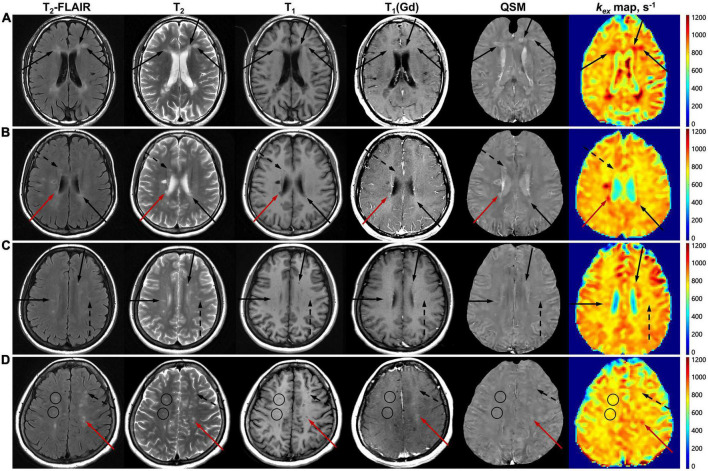
Gd^+^ lesions and the three patterns of Gd^–^ lesions on multimodal MRI. Images are from Patient No. 20 **(A)**, No. 13 **(B)**, No. 11 **(C)**, and No. 27 **(D)**, whose disease duration was 0.5, 1, 2, and 8 years, respectively. Gd^+^ lesions are indicated with red arrows **(B,D)**. Pattern I of Gd^–^ lesions, which are *k*_*ex*_^+^ and QSM^+^, are indicated with black arrows **(A–C)**. Pattern II of Gd^–^ lesions, which are either *k*_*ex*_^+^ and QSM^–^, or *k*_*ex*_^–^ and QSM^+^, are indicated with black dashed arrows **(B–D)**. Pattern III of Gd^–^ lesions, which are *k*_*ex*_^–^ and QSM^–^, are indicated with black circles **(D)**.

52.5% (169/322) of MS lesions did not exhibit Gd enhancement. 76.9% (130/169) of Gd^–^ lesions showed *k*_*ex*_ elevation on the *k*_*ex*_ map and 85.7% (145/169) of Gd^–^ lesions showed hyperintensity on QSM. Of all Gd^–^ lesions, three radiological patterns covering four subtypes of MS lesions were recognized according to the lesion manifestation on the *k*_*ex*_ map and QSM images (Figures 3, 4).

**FIGURE 5:**
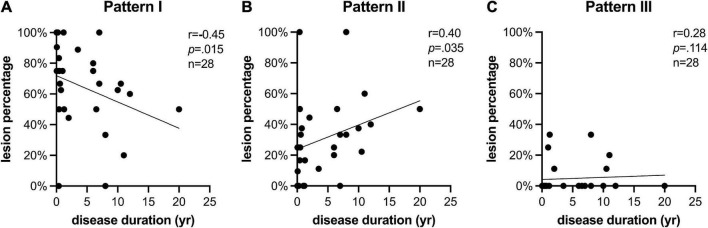
*k*_*ex*_ multimodal MRI pattern is related to disease duration. The percentage of lesions identified as **(A)** Pattern I (which are *k*_*ex*_^+^ and QSM^+^); **(B)** Pattern II (which are either *k*_*ex*_^+^ and QSM^–^, or *k*_*ex*_^–^ and QSM^+^); and **(C)** Pattern III (which are *k*_*ex*_^–^ and QSM^–^) changed with the disease duration of the case. Cases with long disease duration showed smaller percentage of Pattern I but larger percentage of Patten II in Gd^–^ lesions than cases with short disease duration.

Pattern I (*k*_*ex*_^+^ and QSM^+^, *n* = 114) took up the largest proportion of all Gd^–^ lesions, while Pattern III (*k*_*ex*_^–^ and QSM^–^, *n* = 8) took up the smallest. Pattern II (only *k*_*ex*_^+^ or QSM^+^, *n* = 47) was composed of two subtypes: Pattern IIa (*k*_*ex*_^+^ and QSM^–^, *n* = 16) and Pattern IIb (*k*_*ex*_^–^ and QSM^+^, *n* = 31) (Figure 3). The percentage of lesions of each pattern varied among cases (Figure 5). The percentage of Pattern I lesions in each patient was negatively correlated with disease duration (*r* = –0.45, *p* = 0.015, Figure 5A), while the percentage of Pattern II lesions was positively correlated with disease duration (*r* = 0.38, *p* = 0.046, Figure 5B).

**FIGURE 6:**
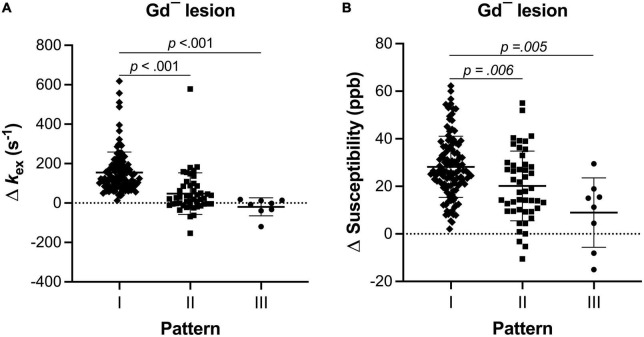
The level of inflammation indicator of each pattern of Gd^–^ MS lesions was different (Kruskal–Wallis test with Dunn-Sidàk correction). Compared with Pattern II and Pattern III, Pattern I also showed higher *k*_*ex*_ elevation **(A)** and higher susceptibility elevation **(B)**.

Different levels of *k*_*ex*_ and susceptibility elevation were also observed within the three patterns (Figure 6). The Pattern I lesions presented higher *k*_*ex*_ and susceptibility elevation (155.0 ± 103.7 s^–1^ and 28.19 ± 12.87 ppb) than both Pattern II (47.8 ± 105.5 s^–1^ and 20.16 ± 14.67 ppb, *p* < 0.001) and Pattern III (–19.61 ± 45.74 s^–1^ and 8.93 ± 14.59 ppb, *p* < 0.01) lesions.

## Discussion

In the present study, we further classified Gd^–^ lesions into three sub-patterns with different levels of *k*_*ex*_ and susceptibility elevation. Both *k*_*ex*_ and susceptibility elevation of Pattern I were higher than Pattern II. Furthermore, the patient with longer disease duration tended to have a lower percentage of lesions with Pattern I.

Multiple pathological processes occur during the course of MS, but the disruption of the BBB (Gaitan et al., 2011) the iron redistribution, and the oxidative damage (Dunham et al., 2017) are among the crucial inflammatory mechanisms. Assessing their correlation could be valuable for gaining a deeper understanding of lesion evolution. In the present study, three MR imaging sequences (Gd-enhanced, QSM, and *k*_*ex*_ MRI) were used to compare the structural damage or the molecular changes from these three mechanisms, respectively. Gd enhancement usually suggests acute inflammation and consequential BBB disruption. The degree of axon demyelination and iron deposition could be reflected by susceptibility, while *k*_*ex*_ has been demonstrated to be able to reflect the overproduction of ROS due to oxidative stress (Tain et al., 2019).

On such premises, Patterns I & II could be regarded as radiologically active for at least one sequence suggesting the existence of inflammation, while Pattern III (which are Gd^–^, *k*_*ex*_^–^, and QSM^–^) might be considered radiologically inactive because inflammation was not detected by either QSM or *k*_*ex*_ MRI. Although the inconsistency of *k*_*ex*_ elevation and QSM hyperintensity did exist, over 70% of all MS lesions were consistently positive on both sequences, which is likely because iron can also serve as the catalyst for the Fenton reactions producing ROS (Tain et al., 2018). This finding suggests that the inflammation due to iron redistribution and oxygen burst can persist regardless of BBB repair. Therefore, interpreting all Gd^–^ lesions as inactive is inaccurate.

Ring enhancement, compared with nodular enhancement, has been considered to result in longer and more severe tissue damage (Davis et al., 2010). Patients with ring-enhancing lesions were also reported to be to reach a EDSS of 4.0-6.0 in shorter time (Llufriu et al., 2010). Ring hyperintensity on QSM, as a sign of iron-containing cells surrounding the lesion edge, has also been related to more severe demyelination and chronic lesion activity/expansion (Absinta et al., 2013; Zhang et al., 2016a). Of the Gd-enhanced lesions (the acute lesions), lesion *k*_*ex*_ elevation was higher in either ring Gd^+^ lesions or ring QSM^+^ lesions than in nodular lesions, which implies that *k*_*ex*_ elevation is positively correlated with the severity of lesion inflammation though further pathologic evidence based on animal experiments are pending for verification. Endogenous ROS of MS is reported to come from the oxygen burst in microglia, inducing mitochondrial dysfunction and subsequent histotoxic hypoxia (Fischer et al., 2012). This further results in energy deficiency and ionic imbalance in oligodendrocytes, axons, and neurons (Mahad et al., 2015). When the respiratory burst leads to cellular degradation, the ferrous iron released from microglia and macrophages may in turn amplify inflammatory oxidative injury and neurodegeneration (Haider, 2015). The mild positive correlation between susceptibility and *k*_*ex*_ elevation we found may just reflect the interplay of iron content and ROS in MS lesions.

Based on the potential mutual reinforcement of iron deposition and oxygen damage, Pattern I is more likely to be inflammatory than Pattern II. For patients with relapsing-remitting courses, the inflammatory status of lesions might not always stay at the same level; instead, it could drop from a higher level to a lower one. The change in the percentage of Pattern I lesions with disease duration might reflect such evolution of chronic inflammation. As for the change in the percentage of Pattern II lesions, it may represent the combination of two different evolutionary trends, since Pattern II consists of two subtypes: IIa (which are *k*_*ex*_^+^ and QSM^–^) and IIb (which are *k*_*ex*_^–^ and QSM^+^). Comparing to the other two patterns, a relatively larger standard deviation of *k*_*ex*_ elevation in Pattern II might also reflect the highly heterogenous nature of it. In the present study, the two subtypes were integrated into one pattern because of the possibly similar level of inflammation, which was lower than Pattern I but higher than Patter III. The difference between *k*_*ex*_ MRI and QSM reflects their differences in contrast mechanism. Nevertheless, attributing *k*_*ex*_ MRI contrast to oxidative stress and ROS production and comparing iron-induced inflammation requires further validation with biochemical analyses.

Lesion susceptibility elevation has generally been reported to remain relatively stable for months or years (Stuber et al., 2016), but lesion *k*_*ex*_ elevation may possess a more volatile trait since if the balance of pro- and antioxidants has not been achieved, ROS may not cease to fluctuate. Unlike the other two imaging sequences, the more susceptible ROS production seems to make the *k*_*ex*_ map a more sensitive method for inflammation indication and curative response monitoring. By enhancing the sensitivity and specificity, the combination of *k*_*ex*_ and QSM MRI could help to further stratify the lesion inflammatory status and detect smoldering lesions after the acute stage. In combination with QSM, *k*_*ex*_ MRI is promising for detection of lesion inflammation or activity.

Clinically, MS patients with a slow or moderate disease course typically receive a moderately effective and very safe medication, whereas patients who demonstrate highly active or rapidly evolving disease are candidates for highly effective but potentially less safe treatment (Thompson et al., 2018b). The time lost in the wait-and-see approach delays highly critical intervention for patients who are later identified as rapidly progressing. Therefore, an improved ability to risk-stratify MS lesion activity with *k*_*ex*_ MRI would help predict prognosis and guide treatment. The clinicians might also utilize non-invasive longitudinal *k*_*ex*_ MRI to monitor the therapeutic efficacy of treatment. For example, a drop of Pattern I percentage in all MS lesions might suggest good treatment response. With an extended lesion classification, a more personalized treatment is facilitated.

This study presents a preliminary exploration of the feasibility of QSM and *k*_*ex*_ MRI in MS lesion categorization based on retrospective, cross-sectional, single-center data. The sample size is relatively small, and the small lesion amount affected the statistical analysis of the two subtypes of Pattern II as well as Pattern III. To conform with *k*_*ex*_ MRI, the image analysis was limited to one slice of image for each case and was also restricted by the spatial resolution of Z-spectral MRI. The errors on lesion registration and kex measurement may exist because there are small lesions which are challenging to differentiate from potential ischemic lesions. Moreover, further validation with pathological studies on preclinical animal models is needed. Research with larger samples, prospective and longitudinal designs, and invasive biochemical validation should be carried out.

In conclusion, the study extended the classification of MS lesions by the combination of three MRI sequences (Gd-enhanced, QSM, and *k*_*ex*_ MRI). Underlying inflammation of Gd^–^ lesions was further visualized by QSM and *k*_*ex*_ MRI, suggesting their utility in assessing the inflammation status of lesions previously considered inactive with Gd-enhanced MRI.

## Data availability statement

The raw data supporting the conclusions of this article will be made available by the authors, without undue reservation.

## Ethics statement

The studies involving human participants were reviewed and approved by the Institutional Review Board of Tongji Hospital, Tongji Medical College, Huazhong University of Science and Technology. Written informed consent for participation was not required for this study in accordance with the national legislation and the institutional requirements.

## Author contributions

HL: analysis and interpretation of data, drafting and revision of the manuscript for content, including medical writing for content, and study concept or design. ZC: processing, analysis and interpretation of data, drafting of the manuscript for content, and including medical writing for content. HY and QC: major role in the acquisition of data and analysis of data. YZ: acquisition of data. MS and SL: revision of the manuscript for content and including medical writing for content. WC: study concept or design, acquisition and interpretation of data, and revision of the manuscript for content. KC: study concept or design, interpretation of data, and revision of the manuscript for content. All authors contributed to the article and approved the submitted version.
