# Supplementary material for: Combining in vivo proton exchange rate (kex) MRI with quantitative susceptibility mapping to further stratify the gadolinium-negative multiple sclerosis lesions
Source: Front Neurosci. 2023 Jan 11;16:1105376. doi: 10.3389/fnins.2022.1105376 (PMC9875136; doi:10.3389/fnins.2022.1105376)
Supplement: Supplementary file 1 [file Table_1.DOCX]

**Supplementary Table 1. Demographic and clinical data**

| **Patient No.** | **Sex** | **Age** | **MRI Exam Amount** | **MRI Exam No.** | **Rough Disease Duration (month)** | **Kurtzke Expanded Disability Status Scale (EDSS)** |
| --- | --- | --- | --- | --- | --- | --- |
|  |  |  |  |  |  |  |
| 1 | F | 18 | 1 | 1 | 0.5 | 6.0 |
| 2 | M | 37 | 1 | 2 | 144 | 3.0 |
| 3 | M | 32 | 4 | 3 | 72 | 3.0 |
|  |  |  |  | 4 | 78 | 1.0 |
|  |  |  |  | 5 | 84 | 1.0 |
|  |  |  |  | 6 | 84 | 1.0 |
| 4 | F | 33 | 2 | 7 | 0.6 | 2.5 |
|  |  |  |  | 8 | 4.6 | 1.5 |
| 5 | M | 34 | 1 | 9 | 126 | 8.5 |
| 6 | F | 49 | 1 | 10 | 12 | 2.0 |
| 7 | F | 31 | 2 | 11 | 120 | 3.0 |
|  |  |  |  | 12 | 120 | 1.5 |
| 8 | F | 22 | 1 | 13 | 132 | 3.5 |
| 9 | M | 27 | 1 | 14 | 72 | 4.0 |
| 10 | F | 32 | 1 | 15 | 6 | 3.0 |
| 11 | F | 31 | 1 | 16 | 24 | 1.0 |
| 12 | F | 31 | 4 | 17 | 0.9 | 3.0 |
|  |  |  |  | 18 | 1.6 | 1.0 |
|  |  |  |  | 19 | 5.2 | 1.0 |
|  |  |  |  | 20 | 15.1 | 1.0 |
| 13 | F | 37 | 1 | 21 | 96 | 1.5 |
| 14 | M | 23 | 1 | 22 | 6 | 3.0 |
| 15 | F | 20 | 1 | 23 | 0.8 | 2.5 |
| 16 | F | 20 | 1 | 24 | 5 | 1.0 |
| 17 | F | 27 | 1 | 25 | 42 | 3.5 |
| 18 | M | 33 | 1 | 26 | 12 | 4.0 |
| 19 | F | 29 | 1 | 27 | 120 | 2.5 |
| 20 | M | 32 | 2 | 28 | 6 | 2.5 |
|  |  |  |  | 29 | 14 | 1.5 |
| 21 | F | 28 | 2 | 30 | 1 | 3.0 |
|  |  |  |  | 31 | 7 | 1.0 |
| 22 | M | 48 | 1 | 32 | 108 | 2.0 |
| 23 | F | 18 | 2 | 33 | 7 | 3.0 |
|  |  |  |  | 34 | 9 | 1.0 |
| 24 | M | 54 | 1 | 35 | 24 | 3.0 |
| 25 | F | 19 | 1 | 36 | 1 | 1.0 |
| 26 | M | 23 | 1 | 37 | 3 | 3.5 |
| 27 | F | 31 | 1 | 38 | 96 | 6.0 |
| 28 | F | 34 | 1 | 39 | 0.6 | 2.0 |
| 19 | F | 54 | 1 | 40 | 240 | 2.0 |
| 30 | F | 32 | 1 | 41 | 0.6 | 1.5 |
