# Supplementary material for: Combining in vivo proton exchange rate (kex) MRI with quantitative susceptibility mapping to further stratify the gadolinium-negative multiple sclerosis lesions
Source: Front Neurosci. 2023 Jan 11;16:1105376. doi: 10.3389/fnins.2022.1105376 (PMC9875136; doi:10.3389/fnins.2022.1105376)
Supplement: Supplementary file 2 [file Table_2.DOCX]

**Supplementary Table 2. Interobserver agreement**

| **Object** | **Criteria** | **Fleiss’ Kappa** | **ICC** | ***p*-value** |
| --- | --- | --- | --- | --- |
| Lesion presence |  |  |  |  |
| Gd enhancement | -/+ | 0.813 | \ | <.001 |
| QSM hyperintensity | -/+ | 0.893 | \ | <.001 |
| *k_ex_* elevation | -/+ | 0.838 | \ | <.001 |
| Lesion shape |  |  |  |  |
| Gd enhancement | nodular/ring | 0.899 | \ | <.001 |
| QSM hyperintensity | nodular/ring | 0.887 | \ | <.001 |
| Quantitative measurement |  |  |  |  |
| ΔSusceptibility | (/ppb) | \ | 0.816 | <.001 |
| Δ*k_ex_* | (/s^-1^) | \ | 0.905 | <.001 |
| ICC, intraclass correlation coefficient; Gd, gadolinium; QSM, quantitative susceptibility mapping; *k_ex_*, proton exchange rate. | | | | |
